# Generation of a recombinant temperature-sensitive influenza D virus

**DOI:** 10.1038/s41598-023-30942-z

**Published:** 2023-03-07

**Authors:** Hiroho Ishida, Shin Murakami, Haruhiko Kamiki, Hiromichi Matsugo, Misa Katayama, Wataru Sekine, Kosuke Ohira, Akiko Takenaka-Uema, Taisuke Horimoto

**Affiliations:** Laboratory of Veterinary Microbiology, Graduate School of Agricultural and Life Sciences, The University of Tokyo, Tokyo, Japan

**Keywords:** Microbiology, Vaccines, Virology

## Abstract

Influenza D virus (IDV) is a causative agent of the bovine respiratory disease complex (BRDC), which is the most common and costly disease affecting the cattle industry. For developing a candidate vaccine virus against IDV, we sought to produce a temperature-sensitive strain, similar to the live attenuated, cold-adapted vaccine strain available against the influenza A virus (IAV). To this end, we produced a recombinant IDV (designated rD/OK-AL) strain by introducing mutations responsible for the adaptation of the IAV vaccine strain to cold conditions and conferring sensitivity to high temperatures into PB2 and PB1 proteins using reverse genetics. The rD/OK-AL strain grew efficiently at 33 °C but did not grow at 37 °C in the cell culture, indicating its high-temperature sensitivity. In mice, rD/OK-AL was attenuated following intranasal inoculation. It mediated the production of high levels of antibodies against IDV in the serum. When the rD/OK-AL-inoculated mice were challenged with the wild-type virus, the virus was not detected in respiratory organs after the challenge, indicating complete protection against IDV. These results imply that the rD/OK-AL might be a potential candidate for the development of live attenuated vaccines for IDV that can be used to control BRDC.

## Introduction

Influenza D virus (IDV), a new member of the family *Orthomyxoviridae*, was initially isolated from pigs with respiratory symptoms in the United States in 2011^1^. Further epidemiological studies demonstrated cattle as the primary host^2,3^, and IDVs circulated in cattle in the American^2–9^, European^10–14^, Asian^15–17^, and African countries^18,19^. Although IDV infection causes mild to moderate respiratory illnesses in cattle, two metagenomic studies conducted on cattle diagnosed with bovine respiratory disease complex (BRDC) have found a positive correlation between IDV infection and BRDC^5,20^. BRDC is the most common and costly disease affecting the cattle industry^21–23^. In addition, antibodies against IDV have been found in pigs^24^, sheep^25,26^, goats^25,26^, horses^27^, dromedary camels^18,28^, deer^29^, and humans^30,31^. These findings indicate that IDVs are distributed globally with multiple animal hosts.

Since IDV might be one of the causative pathogens of BRDC, the development of vaccines for IDV infection is expected to reduce the incidence of BRDC. A research group in the U.S. produced a prototype inactivated vaccine, in which IDV was treated with β-propiolactone and examined its efficacy in cattle. However, this vaccine was not highly effective in protecting against IDV infection^32^. Another group designed a DNA vaccine expressing consensus hemagglutinin-esterase-fusion (HEF) protein of IDV with sequence manipulation. The protective effect of the vaccine was examined against challenges with two lineages of IDVs in a guinea pig model; high vaccine efficacy was observed^33^.

To efficiently induce protective immune effects against respiratory virus, an intranasal spray type live-attenuated vaccine, which can mediate the mucosal immunity to prevent virus infection itself, might be the most desirable dosage form compared to the inactivated vaccine that primarily induces IgG antibody production in the blood. At present, the multivalent modified-live viral vaccine containing attenuated bovine herpesvirus 1, bovine parainfluenza virus 3, and bovine respiratory syncytial virus is commercially available and widely used to control BRDC^23^. Therefore, the attenuated live vaccine could be promising for IDV infection. However, no report has been published examining the attenuated strain of IDV, and genetic mutations that could attenuate the IDV have not been elucidated. The attenuated strains of influenza A virus (IAV) used for the development of live vaccines are the temperature-sensitive, cold-adapted viruses that have been selected by successive passages of the wild-type virus in a low-temperature environment in embryonated chicken eggs or in cell culture^34,35^. These vaccine strains are attenuated due to reduced growth ability at 37 °C, which is the core body temperature in humans. The previous studies indicated that the adaptation of the IAV live vaccine donor strain, A/Leningrad/134/17/57 (H2N2; A/Le17), to cold temperatures was facilitated by mutations in PB2 and PB1 subunits of viral RNA polymerase^36^. Equivalent attenuating effects have been observed when these PB2 and PB1 mutations are introduced into other IAV^36,37^.

In this study, we developed a recombinant IDV strain whose PB2 and PB1 genes were manipulated by introducing mutations responsible for facilitating adaptation of the A/Le17 to cold temperatures using our previously reported reverse genetics system^38^ and examined its temperature sensitivity. Moreover, we evaluated the attenuation phenotype and vaccine efficacy of this recombinant virus by immunization and challenge experiments in a mouse model.

## Results

### Alignment of PB2 and PB1 proteins between IAV and IDV

**Figure 1:**
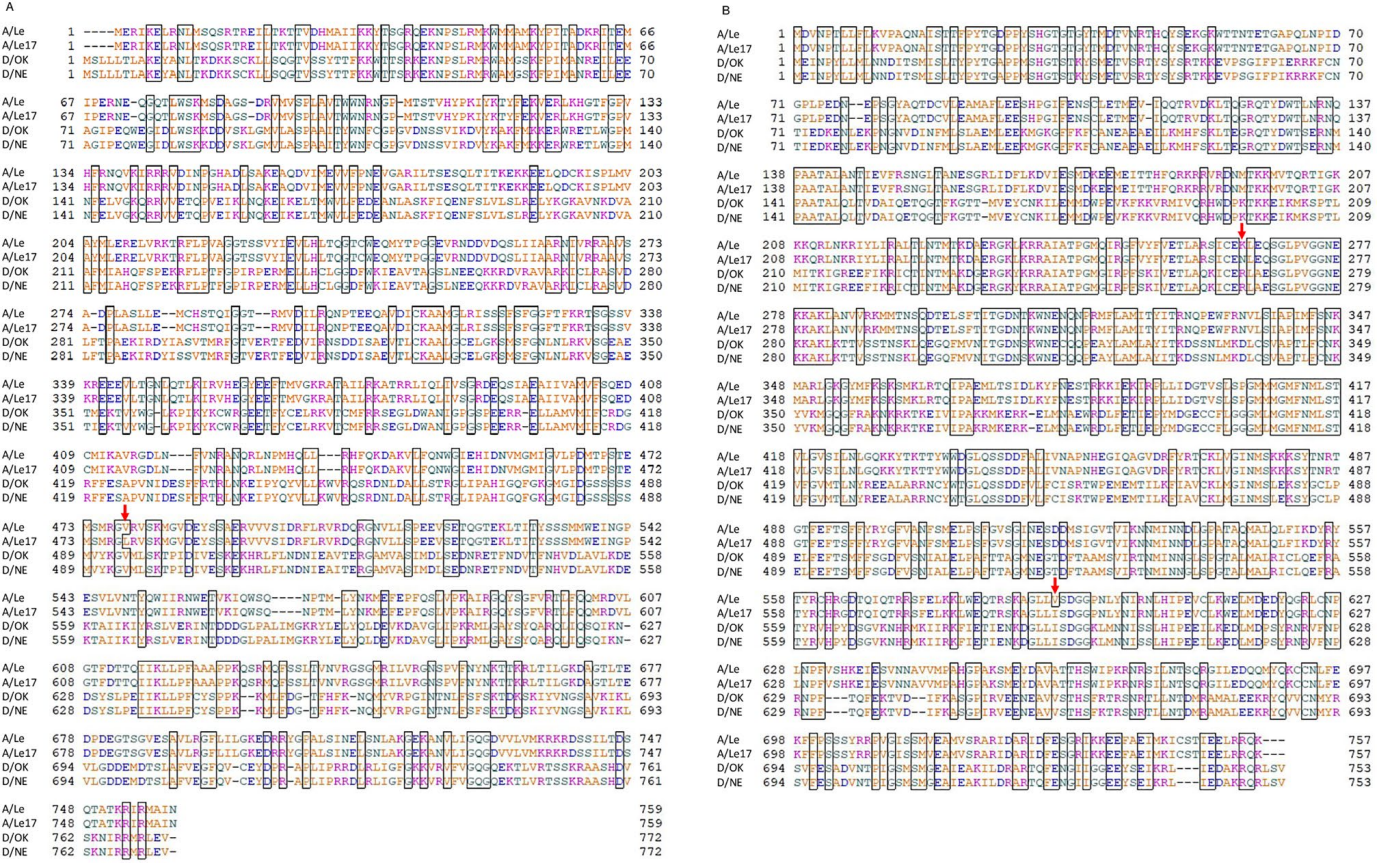
Alignments of PB2 and PB1 protein sequences of influenza A and D viruses. Amino acid sequences of (**A**) PB2 and (**B**) PB1 proteins of the wild type A/Leningrad/134/57 (A/Le), vaccine strain A/Leningrad/134/17/57 (A/Le17; adapted to cold temperatures), D/bovine/Nebraska/9–5/2012 (D/NE), and D/swine/Oklahoma/1334/2011 (D/OK) were aligned. Numbers represent amino acid positions. Hydrophobic, neutral, acidic, and basic amino acids are represented by orange, green, blue, and peach letters, respectively. Consensus amino acids between wild-type IAV and IDV are enclosed in black squares. The amino acid positions responsible for the sensitivity of A/Le17 to high emperatures are indicated by red arrows.

The IAV live vaccine donor strain A/Le17 was generated after 20 sequential passages of the wild-type A/Leningrad/134/57 (A/Le) in chicken embryonated eggs performed at 32 °C and additional 17 passages performed at 25 °C^35,36^. The previous analysis indicated that the high temperature-sensitive, cold-adaptation phenotype in the A/Le17 was primarily facilitated by amino acid mutations V478L in the PB2 and K265N and V591I in the PB1^36^. The RNA polymerase complex of IDV also comprises PB2 and PB1 proteins and possesses structural and functional similarities to IAV^39^. To search for equivalent positions of temperature-sensitive mutations of IAV in IDV, we aligned amino acid sequences of PB2 and PB1 proteins between IAV and IDV (Fig. 1). We postulated that position 478 of A/Le17 PB2 can correspond to position 494 of IDV PB2. Both D/swine/Oklahoma/1334/2011 (D/OK; OK clade) and D/bovine/Nebraska/9-5/2012 (D/NE; 660 clade), belonging to two major clades, possess valine, same as the parent A/Le of A/Le17 (Fig. 1A). Although IDV strains of Y2019 clade possess valine as well, those of Y2016 clade possess isoleucine at this position (data not shown). Similarly, we postulated that positions 265 and 591 of A/Le17 PB1 can correspond to positions 267 and 592 of IDV PB1, respectively; both IDV strains and other clade strains possess arginine at position 267, whereas A/Le possesses lysine at position 265 (Fig. 1B). Interestingly, IDV strains possess isoleucine at position 592, as does A/Le17 at position 591. Taken together, we proposed that PB2 V494L and PB1 R267N mutations may facilitate the sensitivity of IDV to temperatures.

### Construction of IDV with equivalent mutations responsible for the temperature sensitivity of A/Le17

**Figure 2:**
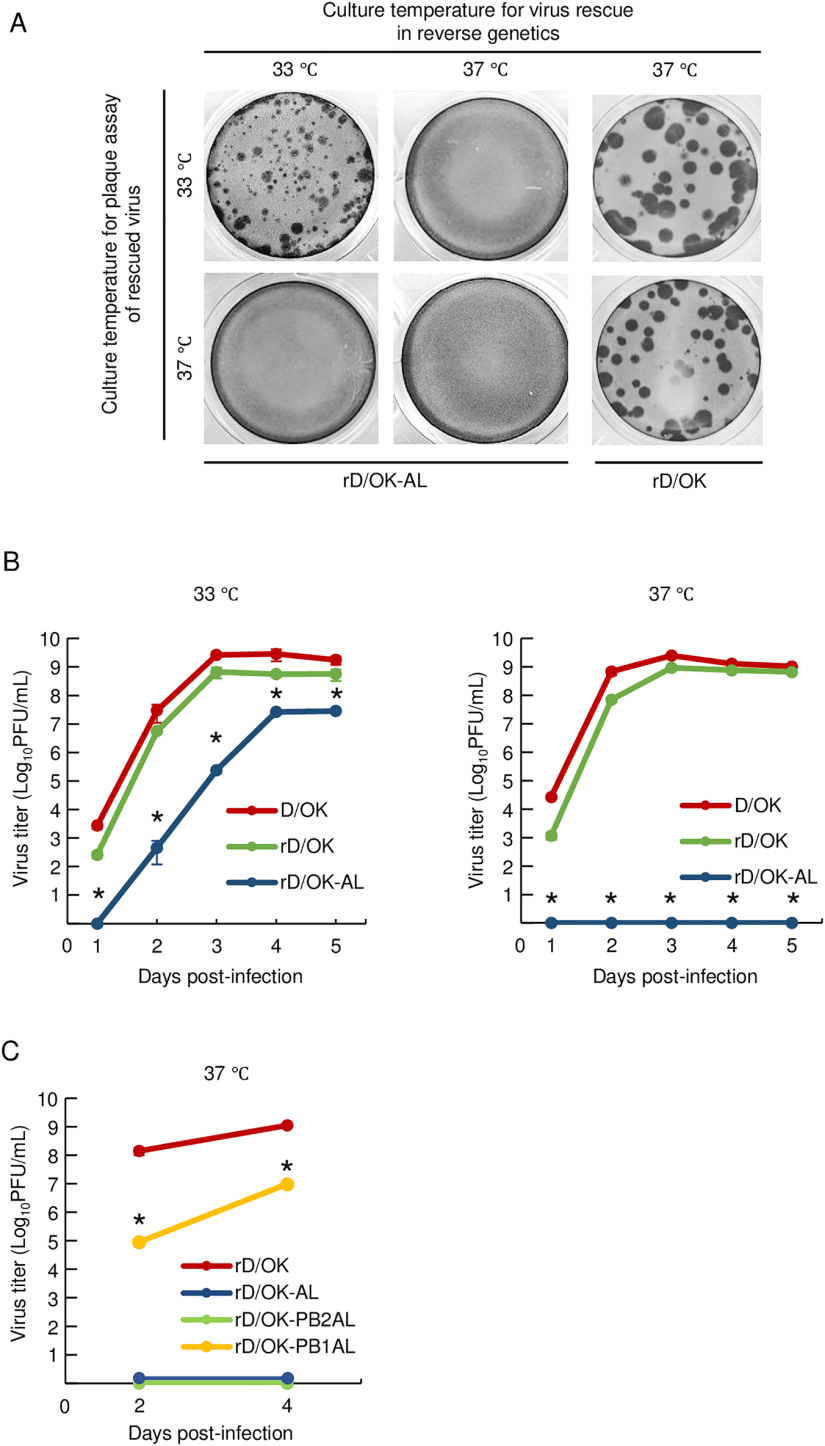
Growth properties of rD/OK-AL at different temperatures. (**A**) The rescue and passage experiments and the plaque assay were performed at 33 °C and 37 °C, respectively. The plaque formation of rescued rD/OK-AL and rD/OK in ST cells is shown at 37 °C for two days or at 33 °C for three days post-infection. Plaques were immunostained with anti-D/OK mouse polyclonal antibody. (**B**) The growth kinetics of rD/OK-AL are shown in ST cells at 33 °C or 37 °C. The wild-type D/OK, rD/OK, and rD/OK-AL were inoculated into cells at a multiplicity of infection of 0.0001 and incubated at 33 °C or 37 °C. Viral titers were determined at 24-h intervals following infection by plaque assay at 33 °C and reported as mean titers with standard deviations (n = 3). This assay’s limit of detection for infectious virus is 10 PFU/mL. The asterisk indicates a significant difference (*p* < 0.05 using two-tailed Student’s *t*-test) between rD/OK and rD/OK-AL at each time-point. (**C**) The growth kinetics of rD/OK-PB2AL and rD/OK-PB1AL are shown in ST cells at 37 °C. The rD/OK, rD/OK-AL, rD/OK-PB2AL, and rD/OK-PB1AL were inoculated into cells at a multiplicity of infection of 0.0001 and incubated at 37 °C. Viral titers were determined at 48-h intervals following infection by the plaque assay at 33 °C and reported as mean titers with standard deviations (n = 3). This assay's limit of detection for infectious viruses is 10 PFU/mL. The asterisk indicates a significant difference (*p* < 0.05 using the two-tailed Student’s *t*-test) between rD/OK and rD/OK-PB1AL at each time-point.

As a strategy for selecting a temperature-sensitive IDV mutant, we sought to generate a recombinant IDV with PB2 V494L and PB1 R267N. We constructed vRNA synthesis plasmids for PB2 and PB1 segments with respective mutations and then generated the recombinant virus via reverse genetics. Because these mutations are expected to attribute temperature sensitivity to the recombinant virus, we performed reverse genetics protocols with cell cultures at 33 °C or 37 °C. The recombinant virus was developed when all procedures with cell cultures were performed only at 33 °C. We designated this rescued recombinant virus as rD/OK-AL. In contrast, no virus was rescued under experimental conditions at 37 °C. The virus rescued at 33 °C did not grow at 37 °C (Fig. 2A), suggesting that the recombinant virus might be sensitive to high temperatures.

### Temperature sensitivity of recombinant rD/OK-AL

To confirm the temperature sensitivity of the rD/OK-AL, we analyzed its growth kinetics in swine testis ST cells at different temperatures. The rD/OK-AL grew only at 33 °C but not at 37 °C (Fig. 2B), confirming its sensitivity to high temperatures. At a temperature of 33 °C, although rD/OK-AL showed lower and slower growth compared to the wild-type D/OK, the corresponding growth was considerably high with a peak titer of more than 10^7^ PFU/mL. These data suggest that rD/OK-AL acquired sensitivity to high temperatures and retained high growth properties at a lower temperature. To evaluate the role that either PB2 V494L or PB1 R267N plays in high-temperature sensitivity, we rescued viruses with each single mutation (namely rD/OK-PB2AL or rD/OK-PB1AL, respectively) at 33 °C and examined their growth properties at 37 °C. The rD/OK-PB1AL did not grow at 37 °C. In contrast, the rD/OK-PB1AL grew at a significantly lower rate than rD/OK (Fig. 2C). These data suggest that PB2 V494L played a vital role and PB2 V494L had a supportive role in the acquisition of high-temperature sensitivity.

### Pathogenicity of rD/OK-AL in mice

**Figure 3:**
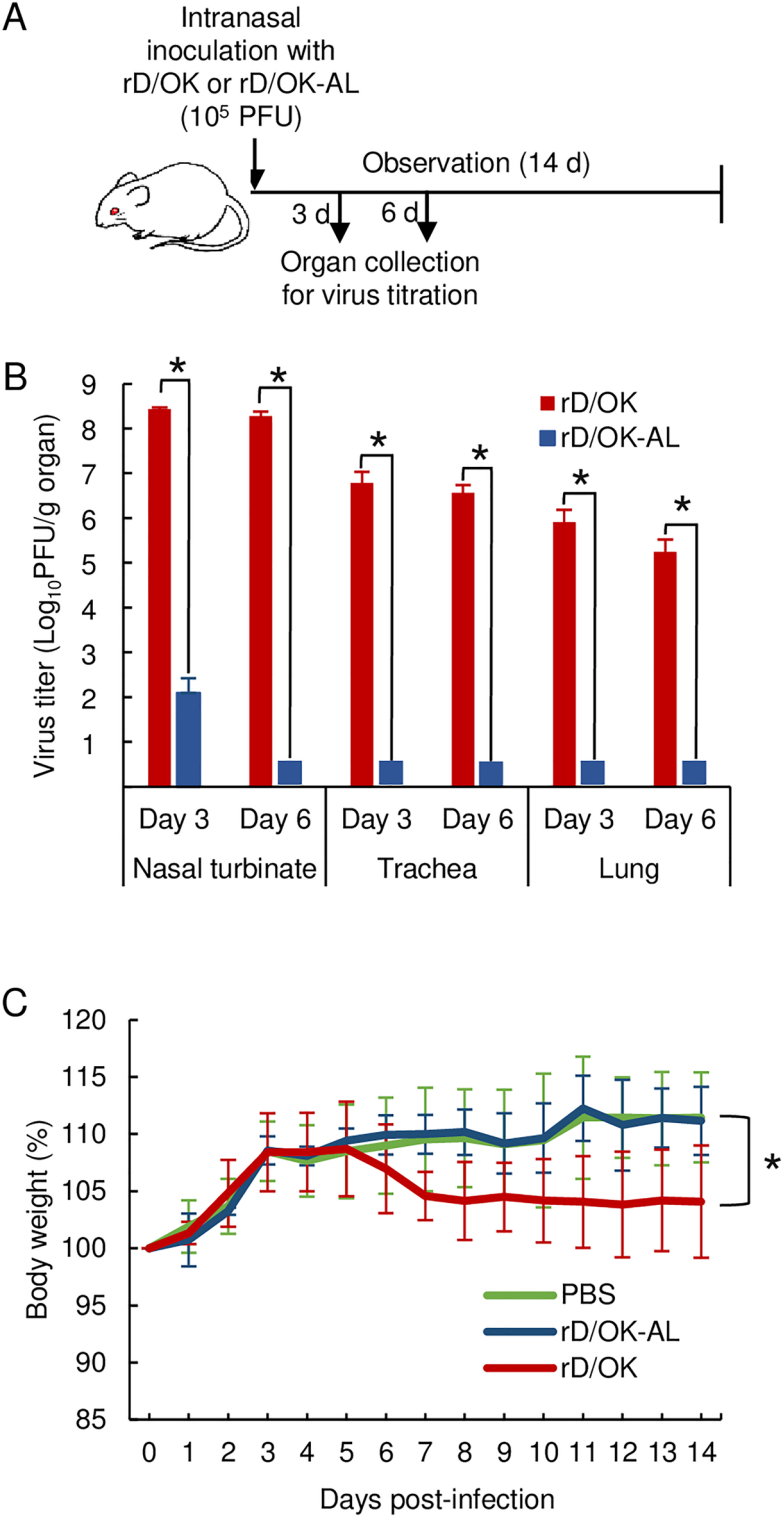
Pathogenicity of rD/OK-AL in mice. (**A**) Schematic presentation of the experimental flow. Respiratory organs containing nasal turbinates, the trachea, and the lungs of mice that were intranasally inoculated with 10^5^ PFU of rD/OK or rD/OK-AL were collected on days three and six following inoculation for viral titration by plaque assay. (**B**) Titers were measured in the organs of rD/OK or rD/OK-AL-infected mice and reported as the mean titers with standard deviations (n = 4, respectively). The limit of detection for infectious virus by this plaque assay is 10^2^ PFU/g. The asterisk indicates a significant difference (*p* < 0.05 by two-tailed Student’s *t*-test) between rD/OK and rD/OK-AL. (C) The kinetics of the body weight of virus-infected or PBS-inoculated mice (n = 5; control) were recorded for 14 days, as reported by changes in percentages based on the values calculated before virus inoculation, which were represented as 100%. The asterisk indicates a significant difference (*p* < 0.05; two-way ANOVA) between rD/OK and rD/OK-AL.

To investigate if the temperature-sensitivity of rD/OK-AL in cell cultures may mediate a change in pathogenicity, we inoculated mice intranasally with 10^5^ PFU of rD/OK-AL or wild-type rD/OK for comparison (Fig. 3A). The rD/OK-AL strain was detected only in nasal turbinates after three days following inoculation and not in the trachea and lungs, whereas rD/OK was detected at high titers in all respiratory organs both after three and six days following inoculation (Fig. 3B). These findings can be possibly attributed to the temperature-sensitivity of the rD/OK-AL because body temperatures are lower in the nasal turbinates than those in the trachea and lungs. In addition, the rD/OK-AL-inoculated mice showed no loss in body weight and kinetics similar to those of the PBS-inoculated control mice during the observation period. In contrast, mice inoculated with the wild-type virus mice showed a trend of weight loss after five days following inoculation (Fig. 3C). Collectively, these results indicate that rD/OK-AL might be highly attenuated in mice.

### Immunogenicity of rD/OK-AL in mice

**Figure 4:**
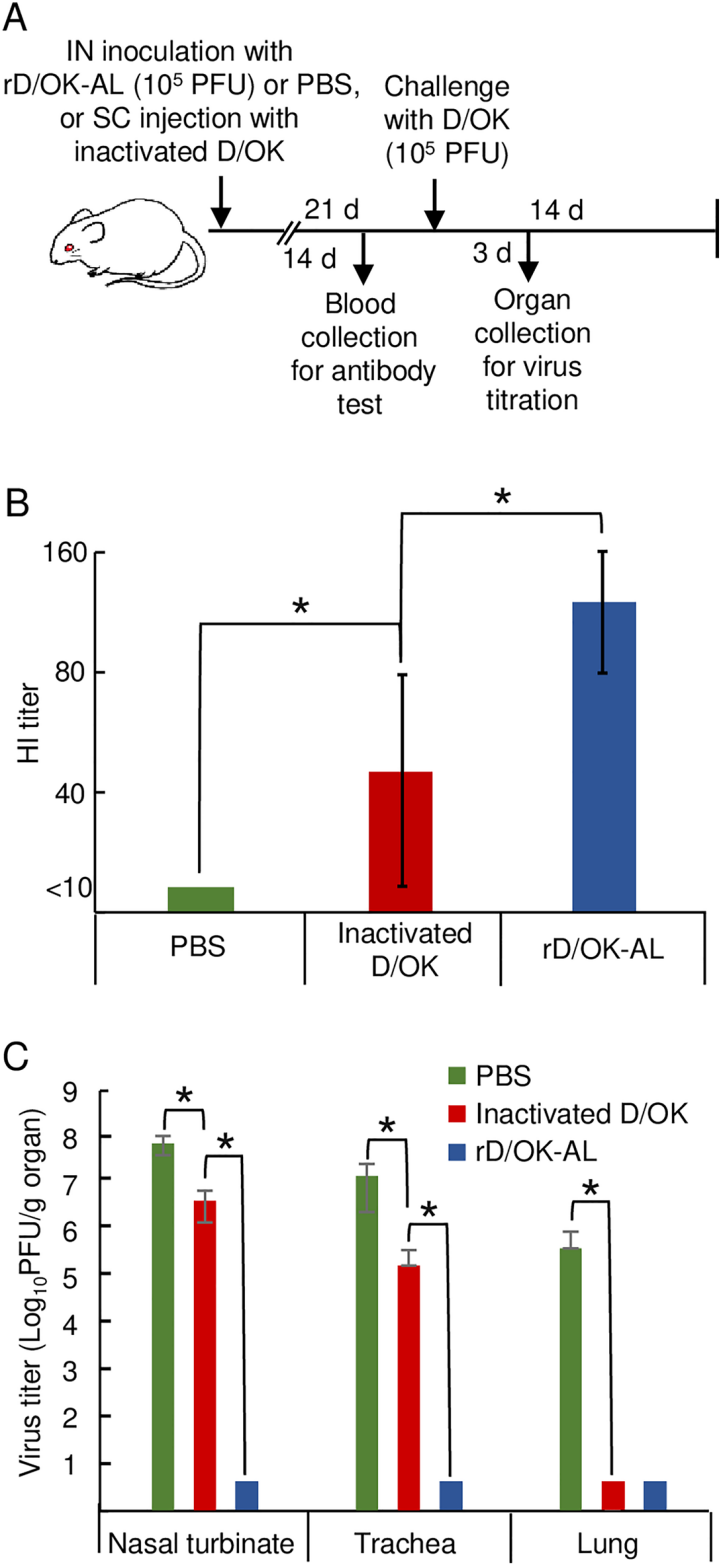
Immunogenicity and protective effect of rD/OK-AL in mice. (**A**) Schematic presentation of the experimental flow. Mice were intranasally (IN) inoculated with rD/OK-AL (10^5^ PFU) or PBS (as a control), or subcutaneously (SC) injected with inactivated D/OK (3.0 µg). Blood was collected from immunized mice for antibody titration by HI test on day 14. The immunized mice were intranasally challenged with wild-type D/OK (10^5^ PFU) on day 21 and then respiratory organs containing nasal turbinates, the trachea, and the lungs of these mice were collected on days three and six following inoculation for determining viral titrates by plaque assay. The clinical signs of the challenged mice were examined over the period of 14 days following the challenge. (**B**) HI antibody titers in serum were determined on day 14 following immunization with rD/OK-AL, inactivated D/OK, or PBS and reported as the mean titers with standard deviations (n = 4, respectively). The asterisk indicates a significant difference (*p* < 0.05 by Kruskal–Wallis test) between rD/OK, rD/OK-AL, and PBS. (**C**) Virus titers were measured in the organs of challenged mice after three days following the challenge and reported as mean titers with standard deviations (n = 4 for each immunized group). The limit of detection for infectious virus by the assay is 10^2^ PFU/g. The asterisk indicates a significant difference (*p* < 0.05 by Kruskal–Wallis test) between rD/OK, rD/OK-AL, and PBS.

To investigate the vaccine potential of rD/OK-AL, we examined its immunogenicity in mice. Mice in each group (n = 3) were intranasally inoculated with either rD/OK-AL (10^5^ PFU) or subcutaneously injected with formalin-inactivated D/OK (3.0 µg) for comparison. Sera were collected on day 14 from mice after administration to detect hemagglutination-inhibition (HI) antibodies against IDV (Fig. 4A). The production of HI antibodies was induced in mice inoculated with rD/OK-AL in higher titers (1:80–1:160) compared to that in mice injected with inactivated D/OK (1:40–1:80) (Fig. 4B). These results suggest that rD/OK-AL might elicit a robust immunogenic response in mice when inoculated intranasally.

### Protective effect of rD/OK-AL against IDV in mice

To examine the protective effects of rD/OK-AL against IDV infection, we challenged mice inoculated with rD/OK-AL or those injected with inactivated D/OK intranasally with wild-type D/OK (10^5^ PFU) after 21 days following administration and examined viral titers in respiratory organs three days after the challenge. No virus was detected in all respiratory organs of rD/OK-AL-inoculated mice whereas high titers of viruses were detected in nasal turbinates and the trachea of mice injected with inactivated D/OK; however, no virus was detected in the lungs unlike control mice injected with PBS that showed high titers of viruses in all organs (Fig. 4C). These results demonstrated that rD/OK-AL-inoculated mice acquired more potent protective immunity against the wild-type virus compared to mice injected with inactivated D/OK, suggesting that rD/OK-AL may possess high vaccine developmental potential for protecting against IDV infection.

## Discussion

Reverse genetics, which allows the artificial development of various mutants, is a powerful tool for the development of recombinant vaccine viruses. The well-designed gene-manipulated recombinant viruses are expected to be used as seed viruses for inactivated vaccines or in attenuated live vaccines either in an emergency or in the future. For instance, any HA/NA reassortants with internal RNA segments of master seed viruses that are adapted to cold temperatures could be robustly generated for the development of human seasonal IAV vaccines^36,40,41^. In addition, candidate vaccines against H5, H7, or H9 avian IAVs have been generated by reverse genetics as a preparation for future pandemics^42–45^. In this study, we applied this technology to develop a candidate vaccine for IDV for reducing the incidence of BRDC which is the most common and costly disease affecting the cattle industry globally. Here, we successfully generated a high temperature-sensitive IDV (rD/OK-AL) strain by introducing equivalent mutations into PB2 and PB1 proteins, which are responsible for temperature-sensitivity and adaptation of IAV live vaccine strain (A/Le17) to cold temperatures^38^. The rD/OK-AL strain demonstrated attenuated phenotype, high immunogenicity, and complete protective effect against IDV infection in a mouse model. These findings indicated a high potential of the rD/OK-AL as a live attenuated IDV vaccine virus in cattle and other livestock.

Theoretically, intranasal spray type live vaccines are more effective against respiratory viral diseases than vaccines administered via other routes, such as intramuscular or subcutaneous injections of inactivated vaccines. Live vaccines administered intranasally could elicit a mucosal immune response that directly block viral infection in respiratory tissues and cells as well as enhance cellular immunity. For live vaccines, high temperature-sensitive viruses are ideally used because they are attenuated due to the lack of growth in lower respiratory organs, such as the lungs. So far, no report has been published examining high temperature-sensitive strains of IDV. In contrast, a couple of temperature-sensitive, cold-adapted strains of IAV have been developed as live vaccines. In IAV live vaccine developed using the A/Le17 strain, PB2 V478L and PB1 K265N and V591I are mutations responsible for its temperature-sensitivity^35,36^. Although precise mechanisms have not been elucidated, the amino acid changes at position 478 in the cap-binding region of PB2 and at positions 265 and 591 in the RNA-binding region of PB1^35,46^ may presumably alter the structural and functional interactions between PB2 or PB1 and viral or host factors at higher temperatures. Notably, these mutations could mediate a similar phenotypic effect in the other IAV^36,37^. Due to functional similarities, the putative structure of functional domains of PB2 and PB1 proteins should be conserved between IAV and IDV, despite their partial identities in amino acid sequences as shown in Fig. 1. Based on this concept, we aimed to develop mutant rD/OK-AL strain by inducing PB2 V494L and PB1 R267N mutations via reverse genetics as a candidate live vaccine for IDV infection. Consequently, this strategy successfully generated a high temperature-sensitive rD/OK-AL. Additional analyses of virus growth with one single mutation suggest that PB2 V494L is mainly and PB1 R267N is synergically required for the D/OK to acquire the *ts* phenotype. Understanding the molecular basis of this observation can contribute towards further design of IDV live vaccines.

During the spread of IAV infection from the upper respiratory tract to the lower respiratory organs, such as the lungs, the damaged tissues induce high levels of cytokines, resulting in systemic symptoms and sometimes severe illness. Therefore, the strains, which cannot replicate in the lungs, can be attenuated for the development of live vaccines^34^. Studies on nasal inoculation of mice with high temperature-sensitive viruses have reported significantly reduced or complete loss of replication in the lungs^40,47^. Although IDV pathogenicity seems different among mouse strains as well as viral strains^48^, since D/OK grows well in respiratory organs of BALB/c mouse model, we used this mouse strain to evaluate the live vaccine potential of rD/OK-AL. We found no viral replication in the lungs of mice after three and six days following inoculation with rD/OK-AL. However, minor viral replication was observed in the nose of mice on day three following inoculation, demonstrating that rD/OK-AL acquired high temperature sensitivity, which was facilitated by mutations introduced, under in vivo as well as in vitro settings. Because the core body temperature of cattle, the main host of IDV, is between 38–39 °C, it was hypothesized that rD/OK-AL does not replicate in the lungs of cattle, leading to its attenuated phenotype in this animal.

Although the rD/OK-AL showed weak growth in the nasal turbinate, sufficient immune response to protect against the challenge with wild-type D/OK was observed in animals following the rD/OK-AL inoculation. This was in contrast to subcutaneous injection of the formalin-inactivated D/OK for which immunogenicity may be insufficient to protect against the wild-type virus challenge. An inactivated candidate vaccine incompletely inhibited disease progression in cattle^32^, consistent with our findings corresponding to formalin-inactivated D/OK. Although the inactivated vaccine induces the production of IgG antibodies in sera of immunized animals, which could block viral growth in lungs as shown in our study (Fig. 3C), the intranasal spray type live vaccines would be more effective for protection against IDV infection. Collectively, our results indicate that the rD/OK-AL can be a highly promising candidate strain for the development of an attenuated live vaccine for IDV infection and controlling BRDC. Studies on *ts* phenotypic stability, pathogenicity, immunogenicity including cellular immune response, and protective effect of rD/OK-AL in cattle are required to evaluate its potential for live IDV vaccine.

The IDVs formed five phylogenetical clades, as determined based on the HEF genes^49,50^. Antigenic heterogeneities among some of these clades have been reported^49,51^. Our constructive strategy involving IDVs with high-temperature sensitivity will be applied to develop any viruses with selected HEF segments for vaccines generated by reverse genetics with the common use of internal RNA segments containing PB2 and PB1. This will aid in the vaccine production designed according to the epidemiology of IDV infection in each country. In addition, this type of live IDV vaccine can be included in the widely used live vaccines for BRDC containing bovine herpesvirus type 1, bovine parainfluenza virus type 3, and bovine respiratory syncytia virus, unless there is interference between IDV vaccine candidate and the existing tri-valent BRDC vaccine. This might act as a more effective mixed vaccine for BRDC.

## Methods

### Cells and viruses

Human rectal tumor HRT-18G cells (obtained from ATCC, CRL-11663) and swine testis (ST) cells (obtained from ATCC, CRL-1746) were maintained in Dulbecco’s modified Eagle’s medium (DMEM; Fujifilm Wako Pure Chemical, Osaka, Japan) supplemented with 10% fetal bovine serum (FBS) at 37 °C. D/swine/Oklahoma/1334/2011 (D/OK) (GenBank accession numbers JQ922305-JQ922311) was kindly provided by Dr. B. Hause (Kansas State University). D/OK was propagated in ST cells in Eagle’s minimum essential medium (MEM; Life Technologies/Gibco, Paisley, UK) containing 0.3% bovine serum albumin (MEM/BSA) supplemented with 0.5 µg/mL L-1-tosylamido-2-phenyl chloromethyl ketone (TPCK)-trypsin (Worthington, Lakewood, NJ, USA) and stored at −80 °C.

### Plasmid construction

vRNA-synthetic plasmids (pPol-D/OK-PB2, -PB1, -P3, -HEF, -NP, -M, and -NS) comprising cDNAs of D/OK viral genes between human RNA polymerase I promoter and mouse RNA polymerase I terminator and eukaryotic protein expression plasmids (pCAGGS-D/OK-PB2, -PB1, -P3, and -NP) under the control of the chicken β-actin promoter were used for reverse genetics as described previously^38^. To generate mutant recombinant viruses, vRNA synthesis plasmids (pPol-D/OK-PB2 and -PB1) with PB2 and PB1 segments were modified. We constructed pol-D/OK-PB2-AL by replacing the 494^th^ codon in pPol-D/OK-PB2 from GTG (encoding valine) to CTC (leucine). Similarly, pPol-D/OK-PB1-AL was constructed by replacing the 267^th^ codon of pPol-D/OK-PB1 from AGA (arginine) to AAT (asparagine).

### Reverse genetics

Reverse genetics was performed as previously described^38^, with some modifications. Briefly, 60% confluent HRT-18G cells were transfected with different amounts of 12 plasmids (0.6 µg each of pPol-D/OK-PB2, -P3, and -NP, 0.1 µg each of pPol-D/OK-PB1, -HEF, -M, and -NS, and 1.0 µg each of pCAGGS-D/OK-PB2, -PB1, -P3, and -NP) on a 6-well plate using PEI MAX (Polysciences, Warrington, PA, USA). The DNA samples and 11 µL of transfection reagent (1 µg/µL) were mixed and incubated at 23 °C for 20 min. The mixtures were then added to the cells, followed by incubation at 33 °C or 37 °C_._ At 24 h post-transfection, the supernatants were removed, and cells were washed twice with Opti-MEM (GIBCO BRL) before the addition of 2 mL of Opti-MEM containing 0.3% BSA. The cells were then incubated for an additional five days at 33 °C or 37 °C. TPCK-trypsin (0.5 µg/mL) was added to the collected culture supernatant, and the mixture was inoculated on ST cells for one hour. The cells were washed twice with MEM before addition of 2 mL of MEM/BSA containing TPCK-trypsin (0.5 µg/mL), and incubated at 33 °C or 37 °C. At five days following infection, supernatants were collected, and viruses were titrated in ST cells via the plaque assay.

### Plaque assay

The plaque assay was performed as described previously^38^. Briefly, confluent ST cells on a 12-well plate were inoculated with 0.1 mL of each virus serially diluted in MEM/BSA and incubated for one hour at 33 °C or 37 °C. Cells were then washed with MEM/BSA, covered with 1 mL of MEM/BSA containing TPCK-trypsin (0.5 µg/mL) and 1% Seakem GTG agarose (Lonza Japan, Chiba, Japan), and incubated at 33 °C or 37 °C for two–three days. Subsequently, 30% formalin in PBS (0.5 mL) was added to each well to fix the cells at 4 °C overnight. After formalin and agarose were removed, the cells were washed with PBS and permeabilized with 0.1% Triton X-100 in PBS for 15 min at 23 °C. After blocking with BlockAce (KAC, Hyogo, Japan), the cells were incubated with anti-IDV mouse immune serum for 60 min, biotinylated anti-mouse IgG antibody (#B7264, Sigma) for 30 min, and a complex comprising streptavidin (8 µg/mL) (Fujifilm Wako Chemicals, Miyazaki, Japan) and biotinylated peroxidase (4 µg/mL) (Invitrogen/Thermo Fisher Scientific, Tokyo, Japan) for 30 min. The plaques were visualized using a DAB peroxidase substrate kit (Vector Laboratories, Burlingame, CA, USA) according to the manufacturer’s instructions.

### Pathogenicity in mice

BALB/c mice (four-week-old, female) were purchased from Japan SLC (Shizuoka, Japan). The mice were divided into three groups with nine animals in each group. Mice were anesthetized and inoculated intranasally (20 µL) of rD/OK or rD/OK-AL (1.0 × 10^5^ PFU), respectively. Organs containing nasal turbinates, the tracheas, and the lungs were collected after three and six days following inoculation and homogenized in phosphate-buffered saline (PBS) for virus titration via the plaque assay.

### Inactivation of the virus

Inactivated D/OK virus was prepared for comparative verification of vaccine efficacy. The virus-containing supernatants from ST cells were purified by low-speed centrifugation to remove cell debris and concentrated by ultracentrifugation at 40,000 × *g* for two hours at 4 °C performed using a P19A rotor (Himac, Tokyo, Japan). The concentrated viruses were resuspended in PBS and layered onto a 20%, 30%, 50%, and 60% discontinuous sucrose gradient and ultracentrifuged at 110,000 × g for three hours at 4 °C using a P32ST rotor (Himac). The virus-containing interface between the 30% and 50% sucrose gradient was collected, diluted in PBS, and ultracentrifuged at 110,000 × *g* for three hours at 4 °C using a P32ST rotor. Purified virus pellets were suspended in a small amount of PBS and inactivated with 0.03% formalin for one week.

### Immunogenicity in mice

Three groups of nine BALB/c mice (four-week-old, female) each were anesthetized and inoculated intranasally (20 µL) with rD/OK-AL (1.0 × 10^5^ PFU; 4–8 HA units) or subcutaneously (200 µL in PBS) with inactivated D/OK (3 µg; 4096 HA units), respectively. The dosage of the inactivated D/OK was selected by referring to previous IAV studies^52,53^. The sera were collected after 14 days following inoculation, and hemagglutination-inhibition (HI) assays were performed for measurement of antibodies against the virus.

### Hemagglutination-inhibition assay

The HI assay was performed in a 96-well microplate with a U-shaped bottom, as previously described^17^. Briefly, serum samples collected from mice were treated with receptor destroying enzyme (RDEII) (Denka Seiken, Tokyo, Japan) to eliminate inhibitors of nonspecific hemagglutination. The serum samples were diluted in RDEII in a 1:3 ratio and incubated for 16 h at 37 °C. The samples were incubated in a 56 °C water bath for 30 min. The samples were diluted by two folds in PBS and incubated with four hemagglutinating units of D/OK. After incubation, 0.7% suspension of turkey red blood cells in PBS were added to each sample, followed by incubation for 30 min. The HI titers were determined as the reciprocal of the highest virus dilution showing complete HI.

### Virus challenge in mice

After 21 days following immunization, the mice were inoculated intranasally with 20 µL of wild-type D/OK (1.0 × 10^5^ PFU). After three days following the challenge (day 24), mice (four animals per group; n = 12) were euthanized, and organs containing nasal turbinates, the tracheas, and the lungs were collected for virus titration via the plaque assay.

### Ethics statement

Our animal study protocols were conducted in accordance with the Regulations for Animal Care at the University of Tokyo and was approved by the Animal Experiment Committee of the Graduate School of Agricultural and Life Sciences at the University of Tokyo (approval number P17-107) following the Fundamental Guidelines for Proper Conduct of Animal Experiment and Related Activities in Academic Research Institutions under the jurisdiction of the Ministry of Education, Culture, Sports, Science and Technology in Japan. All animal experiments were reported in accordance with the ARRIVE guidelines.

## Data Availability

The data used to support the findings of this study are available from the author upon request.
